# Optimization of light spectrum and intensity to enhance growth and metabolite profiles in green and purple radish microgreens cultivated in a vertical farming system

**DOI:** 10.1002/jsfa.70447

**Published:** 2026-01-29

**Authors:** Cristian Hernández‐Adasme, Vicente Martínez, Mónica Flores, Teresa Mestre, Antonio Frutos‐Tortosa, Ulises Navarro‐Zapata, Alejandro Martínez‐Moreno

**Affiliations:** ^1^ Centro de Edafología y Biología Aplicada del Segura (CEBAS‐CSIC) Murcia Spain; ^2^ Laboratorio de Genómica Funcional y Bioinformática, Facultad de Ciencias Agronómicas Universidad de Chile Santiago Chile

**Keywords:** vertical farming, crop yield, nutritional quality, secondary metabolites

## Abstract

**BACKGROUND:**

Microgreens have emerged as a promising crop in vertical farming due to their high nutritional value and short growth cycles. Light spectrum and intensity are critical factors influencing biomass production and metabolic activity in plants, particularly in controlled environments such as vertical farming systems. This study aimed to evaluate the combined effects of light spectrum and intensity on the agronomic traits and metabolite profiles of green and purple radish (*Raphanus sativus* L.) microgreens.

**RESULTS:**

Three commercial LED spectra – NS12 (R:B = 1.9), Ph2.1 (R:B = 2.1) and AP673L (R:B = 5.5) – were tested at three intensities (100, 200, and 300 μmol m^−2^ s ^−1^) in a controlled vertical farming system. Growth parameters (yield, dry matter percentage (DMP), leaf area), photosynthetic pigments, sugars, organic acids, and glucosinolates were quantified. The highest R:B ratio (5.5) under the AP673L lamp at 300 μmol m^−2^ s^−1^ significantly enhanced yield and DMP in both cultivars, while the lowest R:B ratio observed in the NS12 lamp at the same intensity maximized sugar and organic acid accumulation in green microgreens. Glucosinolate content was spectrum‐ and cultivar‐dependent: green radish accumulated higher levels under the lowest R:B ratio (NS12), whereas purple radish responded better to the R:B = 2.1 ratio under the Ph2.1 lamp at lower intensities.

**CONCLUSION:**

These results demonstrate that optimal light strategies in vertical farming must be tailored to specific microgreen genotypes and light conditions to simultaneously maximize productivity and nutritional quality. © 2026 The Author(s). *Journal of the Science of Food and Agriculture* published by John Wiley & Sons Ltd on behalf of Society of Chemical Industry.

## INTRODUCTION

The global microgreens market is experiencing significant growth, driven by their recognized health benefits, exceptional nutritional value, and the increasing adoption of vertical farming systems, particularly in urban environments.^1^ Projections estimate that the market will reach $299.45 billion by 2025, with a compound annual growth rate of 5.42%, leading to a valuation of $433.05 billion by 2032 according to microgreens market report.^2^ This expansion highlights the economic viability of microgreens as high‐value crops, particularly for economically disadvantaged communities, due to their low production costs and premium market prices.^3^

Microgreens are young, edible seedlings harvested during early developmental stages, typically between 6 and 21 days after germination, when they have fully expanded cotyledons and, in some cases, the first true leaf.^3^, ^4^ These crops exhibit nutrient concentrations up to 40 times higher than those of their mature counterparts, classifying them as functional foods with potential health benefits.^1^ Among microgreens, members of the Brassicaceae family – such as radish (*Raphanus sativus*) – are particularly valued for their high levels of essential nutrients and bioactive compounds, including carotenoids, phylloquinones, tocopherols, omega‐3 fatty acids, glucosinolates (GLS), and sulforaphane.^5^, ^6^, ^7^

The controlled environments of vertical farming systems allow precise regulation of growth conditions, with artificial lighting playing a pivotal role. Light spectrum, defined as the distribution of energy across visible and non‐visible wavelengths, is a key factor influencing plant physiology and metabolism.^8^ Among the most widely used spectra, blue and red light combinations are particularly effective in driving photosynthesis and optimizing plant morphology.^9^, ^10^ Blue light enhances chlorophyll biosynthesis and promotes compact growth, while red light stimulates biomass accumulation and leaf expansion.^4^, ^11^, ^12^ Additionally, ultraviolet (UV), green, and far‐red light influence metabolic pathways. Moreover, UV radiation enhances pigment synthesis and secondary metabolite accumulation, green light promotes dry matter production and sugar content, and far‐red light contributes to cell elongation and flowering regulation.^10^, ^13^ On the other hand, light intensity is another critical determinant of microgreen growth and metabolic activity. While intensities exceeding 400 μmol m^−2^ s^−1^ enhance biomass production, lower intensities are often associated with increased chlorophyll and carotenoid accumulation in certain species.^14^, ^15^ However, these effects are species‐specific, emphasizing the need for tailored lighting strategies to optimize both growth and metabolite accumulation.^16^, ^17^

GLS – sulfur‐containing secondary metabolites predominantly found in cruciferous vegetables – have attracted significant scientific and industrial interest due to their antioxidant, anti‐inflammatory, and potential anticancer properties.^7^, ^18^ Their biosynthesis is highly responsive to light conditions, with blue, white, and red light differentially affecting GLS accumulation across species.^19^, ^20^ For example, a red‐to‐blue light ratio of 5:1 has been shown to enhance GLS content in broccoli microgreens, while variations in light intensity further modulate their concentrations in a species‐dependent manner.^12^, ^21^

Despite growing research on the effects of light spectrum and intensity on microgreens, their combined impact on agronomic performance and bioactive compound accumulation in radish microgreens remains underexplored. Furthermore, limited information is available on the interactions between blue–red spectra incorporating UV, green, and far‐red wavelengths and how these interactions vary across cultivars. This study addresses these gaps by evaluating the effects of three LED spectra with different R:B ratios (NS12 (R:B = 1.9), Ph2.1 (R:B = 2.1), and AP673L (R:B = 5.5)) at three intensity levels (100, 200, and 300 μmol m^−2^ s^−1^) on growth parameters, photosynthetic pigments, sugars, organic acids, and GLS accumulation in green and purple radish microgreens. The findings provide critical insights into optimizing lighting strategies for enhanced nutritional quality and yield in vertical farming systems, contributing to the development of more sustainable and resource‐efficient agricultural practices.

## MATERIALS AND METHODS

### Study site and plant material

The experiment was conducted in a vertical farming system (dimensions: 12 m × 2.3 m × 2.4 m) located at the CEBAS‐CSIC research facility in Santomera, Murcia, Spain. Seeds from two radishes cultivars – one with green cotyledons and pink stem (*Raphanus sativus* var. *Longipinnatus*) and the other with red cotyledons (*Raphanus sativus* var. Red Jasper) – were obtained from Kings Seeds (Kelvedon, UK).

### Microgreen cultivation

Seeds were sown under light conditions on compostable cellulose film (dimensions: 20 cm × 12 cm) specifically designed for microgreen cultivation (InstaGreen, Barcelona, Spain). These films were placed in black thermosealable plastic trays (23.1 cm × 14.4 cm × 4 cm). The upper trays, perforated to allow capillary action, supported the cellulose films, while the lower trays contained a Hoagland‐type nutrient solution that was absorbed by the films, preventing waterlogging and algae growth. The nutrient solution had the following composition: KNO_3_ (3 mmol L^−1^), Ca (NO_3_)_2_ (2 mmol L^−1^), MgSO_4_ (0.5 mmol L^−1^), KH_2_PO_4_ (0.5 mmol L^−1^), Fe‐EDTA (10 μmol L^−1^), H_3_BO_3_ (10 μmol L^−1^), MnSO_4_ × H_2_O (1 μmol L^−1^), ZnSO_4_ × 7H_2_O (2 μmol L^−1^), CuSO4 × 5H_2_O (0.5 μmol L^−1^), and (NH_4_)_6_Mo_7_O_24_ × 4H_2_O. The pH of the solution was maintained between 5.2 and 5.6 using nitric acid when necessary. During germination, seeds were kept in darkness for optimal sprouting.

### Light treatments

**Figure 1:**
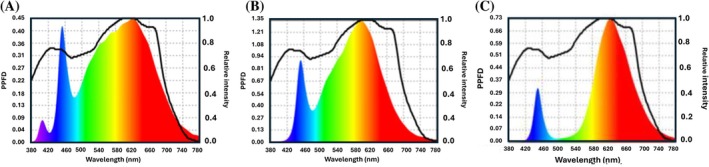
Light spectrum of each light treatment: (A) NS12, R:B = 1.9; (B) Ph2.1, R:B = 2.1; and (C) AP673L, R:B = 5.5. PPFD, photosynthetic photon flux density.

After germination, microgreens were transferred to the shelves (0.4 m × 1.2 m) of a three‐tier rack within the vertical farming system. Each shelf was illuminated by dedicated light‐emitting diode (LED) fixtures, delivering specific spectral compositions and intensities. Three commercial LED treatments with different red‐to‐blue (R:B) ratios were evaluated: (i) NS12, (ii) Phi, and (iii) AP673L, with respective R:B ratios of 1.9, 2.1, and 5.5 (Table 1). Each spectrum was tested under three photosynthetic photon flux densities (PPFDs): 100, 200, and 300 μmol m^−2^ s^−1^. The NS12 and AP673L LEDs were supplied by Valoya (Helsinki, Finland), while Phi LEDs were manufactured by Philips (Amsterdam, Netherlands). Spectral distribution and light intensity were quantified using a Lighting Passport Pro spectroradiometer (Asensetek, New Taipei City, Taiwan) and analyzed with Spectrum Genius Agricultural Lighting software (Asensetek) (Fig. 1). The photoperiod was set to 12 h of light and 12 h of darkness. Environmental conditions were maintained at 22 ± 1 °C with a relative humidity of 65 ± 10%, and continuous air circulation was ensured using ceiling‐ and floor‐mounted fans. The microgreens were harvested 12 days after the light treatments were applied.

**Table 1:** Relative spectral distributions of LED lighting treatments

Light component	Wavelength(nm)	NS12 (%)	Phi (%)	AP673L (%)
Ultraviolet	380–400	1	0	0
Blue (B)	465	19	17	11
Green (G)	532	37	44	19
Red (R)	630	37	36	62
Far Red (FR)	700–800	6	3	8
R:B ratio		1.9	2.1	5.5
R:FR ratio		6.6	12.2	8.1

### Agronomic parameters evaluations

Yield was determined based on the fresh weight (FW) per unit area and expressed in grams per square meter (g m^−2^). At harvest, approximately 50% of the plant material from each tray and replicate was cut and weighed using a digital balance (WLC 2/A2, RADWAG, Radom, Poland) to obtain the fresh weight of the microgreens. The samples were then placed in a drying oven at 70 °C until a constant weight was achieved, at which point the dry weight (DW) was recorded. The dry matter percentage (DMP) was calculated as follows:

$$\mathrm{DMP}=\left(\mathrm{DW}/\mathrm{FW}\right)\;\times \;100$$

Leaf area was measured following the methodology described by Flores *et al*.^22^ Briefly, the total leaf area of the microgreens in each tray and replicate was determined through image analysis. Digital images were processed and analyzed using ImageJ software (v. 1.51.8, NIH, Bethesda, MD, USA).

### Photosynthetic pigments evaluations

Pigments were extracted from 400 mg homogenized microgreens using 4 mL hexane–acetone (3:2) in a magnetic homogenizer (Bullet Blender 5 Gold, Next Advance, Troy, NY, USA) at 4 °C for 1 min. The homogenate was sonicated for 3 min and centrifuged at 10 500 rpm for 15 min at 4 °C. The supernatant was aliquoted into a 96‐well quartz plate, and absorbance was measured at 453, 505, 647, and 663 nm. Chlorophyll *a* (Chla), chlorophyll *b* (Chlb), and carotenoid concentrations were calculated using the following equations:

$$\text{Chla}\;\left(\mathrm{mg}\;L^{-1}\right)=12.25\;\times \;A_{663}–2.79\;\times \;A_{647}$$

$$\text{Chlb}\;\left(\mathrm{mg}\;L^{-1}\right)=21.5\;\times \;A_{647}–5.10\;\times \;A_{663}$$

$$\text{Carotenoids}\;\left(\mathrm{mg}\;L^{-1}\right)=\left(1000\;\times \;A_{470}–1.82\;\times \;\text{Chla}–85.02\;\times \;\text{Chlb}\right)/198$$

where *A* is absorbance. The results were expressed in mg 100 g^−1^ FW.

### Primary metabolites evaluations

#### Total sugars and organic acid concentrations

Lyophilized microgreen samples (100 mg) were ground using a mill (A10 Basic, IKA‐Werke, Staufen, Germany) and extracted with 1 mL of 80% methanol. The extracts were homogenized at 4 °C for 1 min and centrifuged at 11 000 rpm for 5 min at 4 °C, and the supernatant was vacuum‐dried. Metabolite quantification was performed using a Bruker 500 MHz nuclear magnetic resonance (NMR) spectrometer (Bruker BioSpin, Rheinstetten, Germany) equipped with a broadband cryoprobe. Data were processed with Chenomx NMR Suite (v. 8.3, Chenomx, Edmonton, AB, Canada).

#### Total GLS concentration

To determine the total GLS concentration, the methodology of Guijarro‐Real *et al*.^23^ was used, with certain modifications. Briefly, for GLS extraction, 50 mg lyophilized microgreens was mixed with 1 mL of 70% (v/v) methanol. GLS extraction was performed during 30 min at 70 °C, vortexing every 5 min. After heating, the cold extract was centrifuged at 17 500 g for 15 min at 4 °C, and the supernatant was collected. The methanol was then completely removed using a rotary evaporator. The dried sample was dissolved in 1 mL ultrapure water and filtered through a 0.22 μm Millex‐HV13 filter (Millipore, Billerica, MA, USA). GLS identification was performed using a high‐performance liquid chromatography–photodiode array detection–electrospray ionization–tandem mass spectrometry (HPLC‐PAD‐ESI‐MS^*n*^) system (model HPLC1200, Agilent Technologies, Waldbronn, Germany), connected to a Bruker UltraHCT mass detector (Bruker, Bremen, Germany). The ionization conditions were set at a capillary temperature of 350 °C and a voltage of 4 kV. The nebulizer pressure was set at 65.0 psi and the nitrogen flow at 11 L min^−1^. Full mass scan analysis covered the range between *m*/*z* 100 and *m*/*z* 1500. Collision‐induced fragmentation was performed in the ion trap using helium as the collision gas, applying voltage cycles between 0.3 and 2 V. Spectrometric data were acquired in negative ionization mode. MS^*n*^ analysis was performed automatically on the most abundant fragmented ion in the MS^(*n* − 1)^ spectrum. Chromatograms were recorded at a wavelength of 227 nm. GLS quantification was carried out using an HPLC–diode array detection system (HPLC1100, Agilent Technologies), using sinigrin and glucobrassicin (Phytoplan, Diehm und Neuberger GmbH, Heidelberg, Germany) as external standards for aliphatic and indole compounds, respectively. Chromatographic separation was performed on a Luna C18 column (250 mm × 4.6 mm, 5 μm particle size; Phenomenex, Macclesfield, UK). The mobile phase consisted of (A) water with trifluoroacetic acid (99.9:0.1, v/v) and (B) acetonitrile with trifluoroacetic acid (99.9:0.1, v/v). The flow rate was set at 0.8 mL min^−1^, applying a linear gradient that started with 1% B for 5 min, reached 17% B at 15 min (held for 2 min), followed by 25% at 22 min, 35% at 30 min, 50% at 35 min, and finally 99% at 40 min. The total GLS content was determined by summing the different concentrations of the identified GLS compounds, expressing the results per 100 g FW.

### Energy use efficiency and energy economic efficiency

Energy use efficiency (EUE) was calculated from the ratio between the nominal power of each LED lamp provided by the manufacturer and the microgreens fresh weight per tray obtained at the end of the culture period. The electrical consumption of NS‐12 and AP673L lamps for a 12 h photoperiod was 1.34 kW d^−1^ (three lamps × 37.2 W), while for Phi it was 2.80 kW d^−1^ (two lamps × 116.7 W). Therefore, the unit of measurement was kWh kg^−1^. This variable allowed energy consumption to be translated into operating costs when multiplied by the local electricity rate (average rate between 8 a.m. and 8 p.m. on October 6, 2025 = €0.149 kWh^−1^),^24^ thus reflecting the energy economic efficiency (EEE).

### Statistical analysis and experimental design

Data were analyzed using analysis of variance (ANOVA) for each evaluated variable. Mean differences were compared using Fisher's LSD test with a significance level of 5% (*α* = 0.05), considering factor interactions or independent factors where applicable. All statistical analyses were performed using InfoStat software, version 2020e. The experiment followed a completely randomized factorial design (3 × 3), with different R:B ratios under various light spectra (NS12, R:B = 1.9; Ph2.1, R:B = 2.1; AP673L, R:B = 5.5) and intensity (100, 200, 300 μmol m^−2^ s^−1^) as the two independent factors, each with three levels.

## RESULTS

### Agronomic parameters

#### Yield

**Figure 2:**
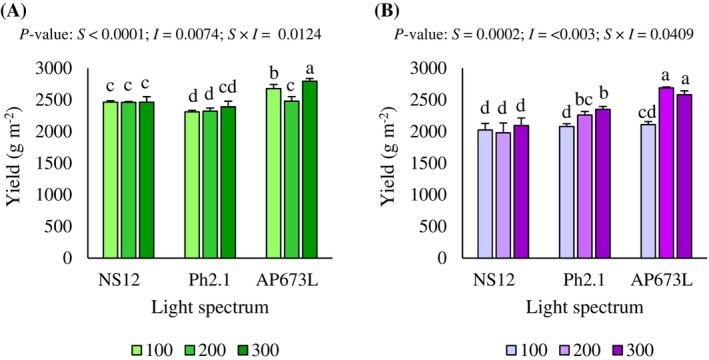
Yield of green (A) and purple (B) radish microgreens as affected by the interaction between LED light spectrum and intensity. Different letters indicate significant differences within each factor or interaction (Fisher's LSD test, *P* < 0.05).

Yield was significantly influenced by the interaction between light spectrum and intensity in both green (*P* = 0.0124) and purple (*P* = 0.0409) radish microgreens (Fig. 2(A),(B)). For green radish microgreens, the yield under the highest R:B ratio (5.5; AP673L lamp) increased progressively with intensity, reaching the maximum value at 300 μmol m^−2^ s^−1^, significantly surpassing all other treatments. In contrast, the R:B ratio of 2.1 (Ph2.1 lamp) promoted the lowest yields, with no significant variations between intensities. Under the lowest R:B ratio (1.9; NS12 lamp), yield remained consistent across all tested intensities, indicating no significant effect of light intensity within this spectrum (Fig. 2(A)). A similar trend was observed for purple radish microgreens. The highest R:B ratio (5.5; AP673L lamp) at 200 and 300 μmol m^−2^ s^−1^ led to significantly higher yields compared to other treatments. Under the lowest R:B ratio (1.9; NS12 lamp), yield decreased significantly compared to the other treatments, and variation in light intensity within this spectrum had no measurable effect on yield. However, under the R:B ratio of 2.1 (Ph2.1 lamp), an increase in yield was observed with intensity, with significant differences between 100 and 200 μmol m^−2^ s^−1^, although the values remained below those obtained with the highest R:B ratio (5.5; AP673L lamp) (*P* < 0.05; Fig. 2(B)).

#### Dry matter percentage

**Figure 3:**
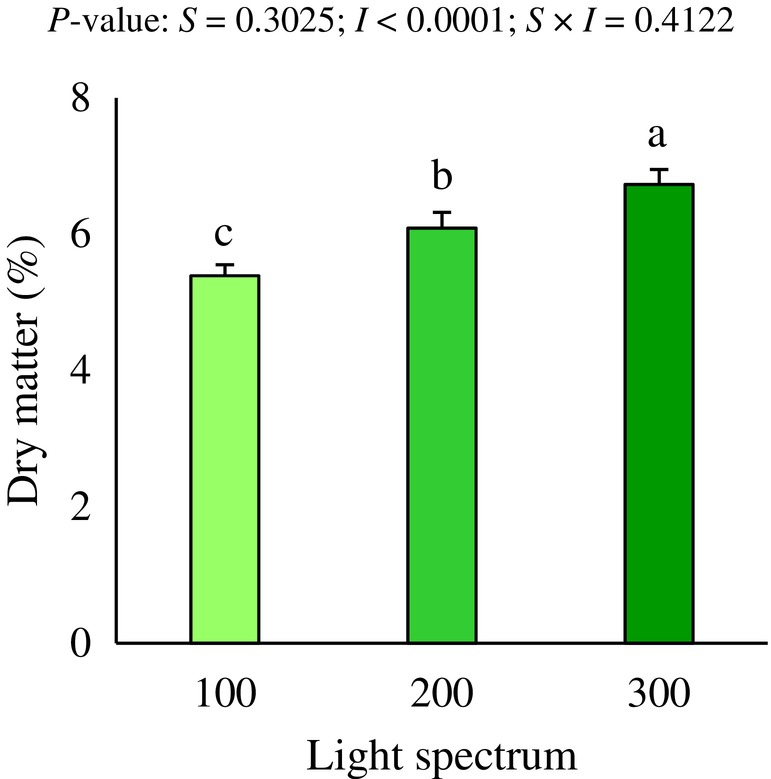
Dry matter percentage of green radish microgreens under the effect of LED light intensity. Different letters indicate significant differences within each factor or interaction (Fisher's LSD multiple range test, *P* < 0.05).

The DMP in green radish microgreens only showed significant differences in the intensity factor (*P* < 0.05; Fig. 3). Specifically, the highest intensity (300 μmol m^−2^ s^−1^) significantly improved the DMP of microgreens relative to medium (200 μmol m^−2^ s^−1^) and low (100 μmol m^−2^ s^−1^) intensities by 10.5% and 24.9%, respectively. Similarly, medium intensity (200 μmol m^−2^ s^−1^) significantly enhanced DMP compared to low intensity (100 μmol m^−2^ s^−1^) by 13.0% (*P* < 0.05; Fig. 3).

**Figure 4:**
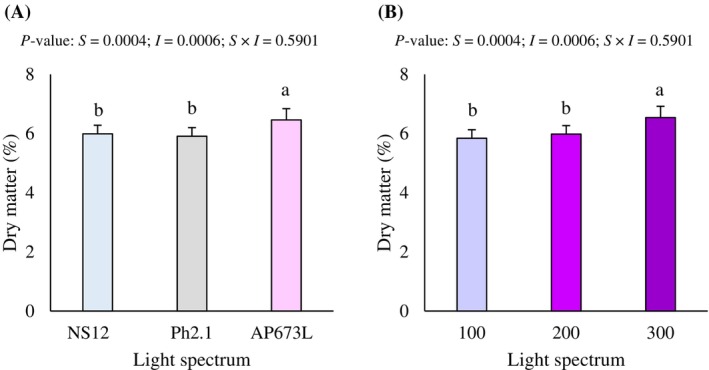
Dry matter percentage of purple radish microgreens under the effect of spectrum (A) and intensity (B) LED light. Different letters indicate significant differences within each factor or interaction (Fisher's LSD test, *P* < 0.05).

Spectrum and intensity factors independently and significantly affected the DMP in purple microgreens (*P* < 0.05; Fig. 4(A),(B)). In particular, the highest R:B ratio (5.5, AP673L lamp) significantly increased DMP compared to lowest R:B ratio (1.9; NS12 lamp) and R:B = 2.1 ratio under Ph.2.1 lamp by 7.8% and 9.3% (Fig. 4(A)), while the highest intensity (300 μmol m^−2^ s^−1^) promoted higher DMP in purple radish microgreens compared to medium (200 μmol m^−2^ s^−1^) and low (100 μmol m^−2^ s^−1^) intensities by 9.4% and 12.0% (*P* < 0.05; Fig. 4(B)).

#### Leaf area

**Figure 5:**
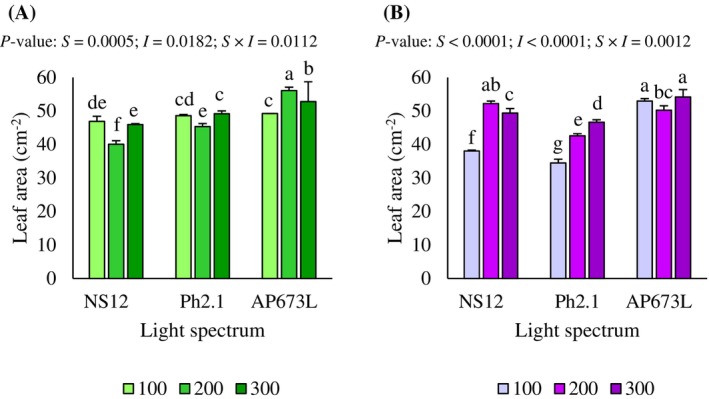
Leaf area of green (A) and purple (B) radish microgreens under the effect of LED light spectrum and intensity interaction. Different letters indicate significant differences within the interaction (Fisher's LSD test, *P* < 0.05).

Leaf area (LA) was significantly influenced by the interaction between light spectrum and intensity in both radish microgreen cultivars (*P* < 0.05; Fig. 5(A)). In green radish microgreens, the highest R:B ratio (5.5; AP673L lamp) at 200 μmol m^−2^ s^−1^ resulted in a significantly larger leaf area compared to 300 and 100 μmol m^−2^ s^−1^ and all other treatments. Under R:B = 2.1 ratio (Ph2.1 lamp), the leaf area was significantly higher, at 300 μmol m^−2^ s^−1^ *versus* 100 and 200 μmol m^−2^ s^−1^. On the other hand, the lowest R:B ratio (1.9; NS12 lamp) at 200 μmol m^−2^ s^−1^ significantly decreased leaf area compared to 100 and 300 μmol m^−2^ s^−1^ and, overall, leaf area values were lowest under NS12 with the lowest R:B ratio (1.9) relative to Ph2.1 and AP673L with R:B ratios equal to 2.1 and 5.5, respectively (*P* < 0.05; Fig. 5(A)). For purple radish microgreens, the highest R:B ratio (5.5; AP673L lamp) at 300 and 100 μmol m^−2^ s^−1^ produced the largest leaf area compared to 200 μmol m^−2^ s^−1^, and all other treatments. Under R:B = 2.1 ratio (Ph2.1 lamp), the leaf area significantly increased with intensity, reaching a maximum at 300 μmol m^−2^ s^−1^ and a minimum at 100 μmol m^−2^ s^−1^. Similarly, an intensity of 100 μmol m^−2^ s^−1^ under the lowest R:B ratio (1.9; NS12 lamp) significantly diminished leaf area compared to 200 and 300 μmol m^−2^ s^−1^ (*P* < 0.05; Fig. 5(B)).

### Photosynthetic pigments

#### Chla and Chlb concentrations

Chla in green radish microgreens was affected only by intensity factor (*P* = 0.015; Table 2). Thus, Chla of green radish microgreens significantly increased under the high (300 μmol m^−2^ s^−1^) and medium (200 μmol m^−2^ s^−1^) intensity *versus* low intensity (100 μmol m^−2^ s^−1^) by 6.5% and 4.3%, respectively. On the other hand, Chla in purple radish microgreens was influenced only by spectrum factor (*P* = 0.0451; Table 2). In particular, the R:B ratio of 2.1 (Ph2.1 lamp) significantly increased Chla compared to the lowest R:B ratio (1.9; NS12 lamp) by 3.6%. Chlb was affected by the interaction of spectrum and intensity factors in green radish microgreens (*P* = 0.0038; Table 2). Overall, a progressive and significant reduction in Chlb concentration was observed as intensity increased from 100 to 300 μmol m^−2^ s^−1^ for the different R:B ratios, although the effect was greater for the R:B = 2.1 ratio (Ph2.1 lamp). Specifically, the lowest R:B ratio (1.9; NS12 lamp) and R:B ratio = 2.1 (Ph2.1 lamp) at 100 μmol m^−2^ s^−1^ significantly increased Chlb compared to 200 and 300 μmol m^−2^ s^−1^, which did not differ statistically from each other but were higher than those of the highest R:B ratio (5.5; AP673L lamp) (Table 2). On the other hand, the Chlb in purple radish microgreens was impacted by spectrum (*P* = 0.0075) and intensity (*P* = 0.0003) factors independently (Table 2). The highest R:B ratio (5.5; AP673L lamp) significantly raised the Chlb compared to R:B ratio = 2.1 (Ph2.1 lamp) and the lowest R:B ratio (1.9; NS12 lamp) by 4.5% and 6.7%, respectively, whereas the highest intensity (300 μmol m^−2^ s^−1^) increased Chlb compared to medium (200 μmol m^−2^ s^−1^) and low intensity (100 μmol m^−2^ s^−1^) by 8.2% and 11.6%, respectively.

**Table 2:** Chlorophyll a concentration, chlorophyll b concentration and carotenoids concentration in green and purple radish microgreens under treatment LED light

Factor	Level	Chlorophyll *a* (mg 100 g^−1^ FW)		Chlorophyll *b* (mg 100 g^−1^ FW)		Carotenoids (mg 100 g^−1^ FW)	
		Green	Purple	Green	Purple	Green	Purple
Spectrum (*S*)	NS12	16.82	18.92b	7.62a	7.10b	57.20	50.80b
	Ph2.1	17.60	19.60a	7.28b	7.13b	55.47	50.93b
	AP673L	17.29	19.46a	7.32b	7.50a	56.17	53.57a
Intensity (*I*)	100	16.58b	19.21	7.94a	6.98b	56.78	50.10b
	200	17.48a	19.37	7.29b	7.18b	56.43	51.39b
	300	17.65a	19.41	7.00c	7.57a	55.63	53.81a
*S* × *I*	NS12 × 100	16.71	19.00	8.13a	7.09	58.92a	51.12c
	NS12 × 200	17.01	18.70	7.34bcd	6.82	58.19a	48.40c
	NS12 × 300	16.75	19.06	7.41bc	7.40	54.48b	52.38bc
	Ph2.1 × 100	16.68	19.33	8.11a	6.96	53.97b	50.00c
	Ph2.1 × 200	17.64	19.33	7.15 cd	7.27	54.73b	50.65c
	Ph2.1 × 300	18.48	20.15	6.59e	7.16	57.71ab	52.15bc
	AP673L × 100	16.36	19.29	7.59b	6.89	57.45ab	49.18c
	AP673L × 200	17.81	20.08	7.37bc	7.45	56.37ab	54.61ab
	AP673L × 300	17.72	19.01	7.01d	8.16	54.70b	56.91a
*P*‐value	*S*	0.0766	0.0451	0.0051	0.0075	0.1913	0.0078
	*I*	0.0150	0.7340	<0.0001	0.0003	0.4612	0.0023
	*S* × *I*	0.2148	0.1799	0.0038	0.0519	0.0366	0.0165

Different letters in the columns within each factor or interaction indicate significant differences (Fisher's LSD test, *P < 0.05).

#### Total carotenoid concentration

Total carotenoid concentration (TCC) was affected by the interaction of spectrum and intensity factors in both cultivars of radish microgreens (*P* = 0.0366 and *P* = 0.0165) (Table 2). In green radish microgreens, a significant decrease in TCC concentration was observed as intensity increased from 100 to 300 μmol m^−2^ s^−1^ for the lowest R:B ratio (1.9; NS12 lamp). A similar trend was observed for the highest R:B ratio (5.5; AP673L lamp) although no significant effect was observed between intensities. Meanwhile, for R:B = 2.1 (Ph2.1 lamp), no significant differences were observed at different light intensities (Table 2). In contrast, purple radishes showed a significant enhancement in TCC as intensity increased under the highest R:B ratio (5.5; AP673L lamp). At the lowest R:B ratios (1.9; NS12 lamp and 2.1; Ph2.1 lamp), a slight increase was observed with the highest intensity (300 μmol m^−2^ s^−1^), but it was not significantly different from 100 and 200 μmol m^−2^ s^−1^ (Table 2).

### Primary metabolites

#### Sugars and organic acid concentration

Total sugar concentration (TSC) was significantly influenced by the interaction between light spectrum and intensity in both radish microgreen cultivars (*P* < 0.001; Table 3). In general, TSC increased progressively under each R:B ratio as intensity augmented, showing a similar trend in both cultivars. In green radish microgreens, the lowest R:B ratio (1.9; NS12 lamp) at 300 μmol m^−2^ s^−1^ resulted in the highest TSC, significantly exceeding 100 and 200 μmol m^−2^ s^−1^, and all other treatments. Similarly, the R:B ratio of 2.1 (Ph2.1 lamp) and the highest R:B ratio (5.5; AP673L lamp) at 300 μmol m^−2^ s^−1^ significantly increased TSC compared to 100 and 200 μmol m^−2^ s^−1^, although the effect was more pronounced under the highest R:B ratio (5.5; AP673L lamp) (Table 3). A similar pattern was observed in purple radish microgreens. The lowest R:B ratio (1.9; NS12 lamp) at 300 μmol m^−2^ s^−1^ also led to the highest TSC compared to 100 μmol m^−2^ s^−1^, 200 μmol m^−2^ s^−1^, and all other treatments, with increases ranging from 5.3% to 142.4%, depending on the treatment comparison. Similarly, the R:B ratio of 2.1 (Ph2.1 lamp) and the highest R:B ratio (5.5; AP673L lamp) at 300 μmol m^−2^ s^−1^ significantly improved TSC *versus* 100 and 200 μmol m^−2^ s^−1^ with the most noticeable effect under the R:B ratio of 2.1 (Ph2.1 lamp). These results highlight the strong influence of light intensity and spectral composition on sugar accumulation, with the lowest R:B ratio (1.9; NS12 lamp) at 300 μmol m^−2^ s^−1^ proving to be the most effective combination for enhancing TSC in both cultivars. The total organic acid concentration (TOAC) was significantly affected by the interaction of the spectrum and intensity factors in green radish microgreens (*P* < 0.0001; Table 3). Particularly, the lowest R:B ratio (1.9; NS12 lamp) at 300 μmol m^−2^ s^−1^ significantly increased TOAC with 100 and 200 μmol m^−2^ s^−1^ and the rest of the treatments. Similarly, with R:B ratios of 2.1 (Ph2.1 lamp) and 5.5 (AP673L lamp), the intensity of 300 μmol m^−2^ s^−1^ significantly promoted TOAC compared to 100 and 200 μmol m^−2^ s^−1^, reaching increases in the range of 74.2–95.8% and 5.0–41.6%, respectively. The results revealed that, across all R:B ratios – 1.9 (NS12 lamp), 2.1 (Ph2.1 lamp), and 5.5 (AP673L lamp) – the lowest light intensity (100 μmol m^−2^ s^−1^) led to the lowest concentration of TOAC. On the other hand, TOAC was significantly influenced by spectrum (*P* = 0.0006) and intensity (*P* < 0.0001) factors independently in purple radish microgreens (Table 3). Specifically, microgreens grown under the highest and lowest R:B ratio (5.5; AP673L lamp and 1.9; NS12 lamp) showed a significantly higher TOAC than microgreens grown under the R:B ratio of 2.1 (Ph2.1 lamp) by 17.1% and 10.0%, respectively. Furthermore, as the intensity increased, the TOAC significantly enhanced. Thus, microgreens grown at the highest intensity (300 μmol m^−2^ s^−1^) significantly increased the TOAC compared to medium (200 μmol m^−2^ s^−1^) and low intensity (100 μmol m^−2^ s^−1^) by 15.8% and 111.6%, respectively. In turn, the medium intensity (200 μmol m^−2^ s^−1^) significantly increased the TOAC compared to the low intensity (100 μmol m^−2^ s^−1^) by 82.7%.

**Table 3:** Total sugar concentration, total organics acid concentration and total glucosinolate concentration in green and purple radish microgreens under treatment LED light

Factor	Level	Total sugars (mg 100 g^−1^ FW)		Total organic acids (mg 100 g^−1^ FW)		Total glucosinolates (mg 100 g^−1^ FW)	
		Green	Purple	Green	Purple	Green	Purple
Spectrum (*S*)	NS12	706.34a	356.27a	147.00a	95.80a	256.30a	212.89ab
	Ph2.1	559.30c	325.74b	99.68b	87.10b	247.30b	219.72a
	AP673L	628.82b	360.04a	94.33b	102.01a	246.70b	208.73b
Intensity (*I*)	100	432.33c	224.04c	72.96c	57.63c	244.39b	225.03a
	200	656.88b	372.20b	124.95b	105.31b	251.71a	215.11b
	300	805.25a	445.80a	143.09a	121.97a	254.21a	201.20c
*S* × *I*	NS12 × 100	486.06f	260.73f	85.69f	62.61	251.62	225.84a
	NS12 × 200	743.34c	337.82e	172.92b	103.16	258.69	208.05b
	NS12 × 300	889.63a	470.25a	182.39a	121.64	258.60	204.77bc
	Ph2.1 × 100	381.66 h	194.03 h	68.89 g	53.41	238.13	237.74a
	Ph2.1 × 200	605.41e	362.67d	95.25e	94.17	251.49	228.25a
	Ph2.1 × 300	690.83d	420.51c	134.90c	113.71	252.29	193.18c
	AP673L × 100	429.27 g	217.37 g	64.30 g	56.87	243.43	211.51b
	AP673L × 200	621.90e	416.10c	106.68d	118.60	244.94	209.04b
	AP673L × 300	835.31b	446.64b	112.00d	130.56	251.75	205.65b
*P*‐value	S	<0.001	<0.001	<0.001	0.0006	0.0028	0.0191
	I	<0.001	<0.0001	<0.0001	<0.0001	0.0062	<0.001
	S × I	<0.001	<0.001	<0.001	0.0681	0.4579	0.0018

Different letters in the columns within each factor or interaction indicate significant differences (Fisher's LSD test, *P < 0.05).

### Total GLS concentration

This variable was significantly affected by spectrum (*P* = 0.0028) and intensity (*P* = 0.0062) independently in green radish microgreens (Table 3). In particular, the lowest R:B ratio (1.9; NS12 lamp) significantly increased total GLS concentration (TGC) relative to the R:B ratio of 2.1 (Ph2.1 lamp) and the highest R:B ratio (AP673L lamp) by 3.8% and 3.9%, respectively. On the other hand, both high (300 μmol m^−2^ s^−1^) and medium (200 μmol m^−2^ s^−1^) intensities significantly improved TGC compared to low intensity (100 μmol m^−2^ s^−1^) by 2.6% and 3.4%, respectively. In purple radish microgreens, TGC was significantly affected by the interaction of spectrum and intensity factors (*P* = 0.0018; Table 3). Particularly, under the lowest R:B ratio (1.9; NS12 lamp) the intensity of 100 μmol m^−2^ s^−1^ significantly improved TGC compared to 200 and 300 μmol m^−2^ s^−1^. Under the R:B ratio of 2.1 (Ph2.1 lamp) both intensities – 100 and 200 μmol m^−2^ s^−1^ – significantly enhanced TGC compared to 300 μmol m^−2^ s^−1^. Meanwhile, with the highest R:B ratio (5.5; AP673L lamp), no significant differences were observed between the intensities. Interestingly, the lowest TGC values were observed under the highest light intensity (300 μmol m^−2^ s^−1^), regardless of the R:B ratio used.

### Energy use efficiency (EUE) and energy economic efficiency (EEE)

Both EUE and EEE were significantly affected by spectrum and intensity independently in both radish microgreen cultivars (Table 4). Specifically, the R:B ratio of 2.1 under the Ph2.1 lamp significantly increased EUE compared to the highest R:B ratio (5.5; AP673L lamp) and the lowest (1.9; NS12 lamp) in green and purple radish microgreens. On the other hand, the lowest intensity (100 μmol m^−2^ s^−1^) enhanced EUE relative to 200 and 300 μmol m^−2^ s^−1^ in both microgreen cultivars. Similarly, EEE showed a similar trend. EEE increased significantly under the R:B ratio of 2.1 (Ph2.1 lamp) compared to the highest (5.5; AP673L lamp) and lowest (1.9; NS12 lamp) R:B ratios in green radish microgreens by 125.4% and 120.7%, respectively. Meanwhile, in purple radish microgreens, the increase was 105.3% and 97.3%, respectively. On the other hand, the lowest intensity of 100 μmol m^−2^ s^−1^ significantly boosted the EEE compared to the intensities of 200 and 300 μmol m^−2^ s^−1^ in green microgreens by 3.6% and 8.5%, respectively. In purple microgreens, this increase was 4.8% and 15.8%, respectively.

**Table 4:** Energy use efficiency and energy economic efficiency in green and purple radish microgreens under treatment LED light

Factor	Level	Energy use efficiency		Energy economic efficiency	
		(kWh kg^−1^)		(€ kg^−1^)	
		Green	Purple	Green	Purple
Spectrum (*S*)	NS12	15.93b	20.09b	2.37b	2.99b
	Ph2.1	35.07a	38.83a	5.23a	5.79a
	AP673L	15.60b	18.9c	2.32b	2.82c
Intensity (*I*)	100	23.09a	28.15a	3.44a	4.19a
	200	22.27b	26.85b	3.32b	4.00b
	300	21.24c	24.31c	3.17c	3.62c
*S* × *I*	NS12 × 100	16.37	20.62	2.44	3.07
	NS12 × 200	16.22	20.07	2.42	2.99
	NS12 × 300	15.21	19.58	2.27	2.92
	Ph2.1 × 100	36.52	41.65	5.44	6.21
	Ph2.1 × 200	34.56	38.21	5.15	5.69
	Ph2.1 × 300	34.13	35.91	5.08	5.35
	AP673L × 100	16.38	20.31	2.44	3.03
	AP673L × 200	16.03	18.47	2.39	2.75
	AP673L × 300	14.4	17.45	2.15	2.6
*P*‐value	*S*	<0.001	<0.001	<0.001	<0.001
	*I*	0.0036	0.0002	0.0036	0.0002
	*S* × *I*	0.5283	0.0841	0.5285	0.0841

Different letters in the columns within each factor or interaction indicate significant differences (Fisher's LSD test, *P < 0.05).

## DISCUSSION

### Influence of light spectrum and intensity on agronomic parameters

The present study demonstrates that both light spectrum and intensity significantly affect the agronomic performance of green and purple radish microgreens, acting through independent and interactive effects. The highest R:B ratio (5.5; AP673L lamp) consistently enhanced yield and dry matter production in both cultivars, particularly at higher intensities (200–300 μmol m^−2^ s^−1^). These findings align with previous reports indicating that red‐enriched spectra, especially when complemented with far‐red (FR) light, stimulate biomass accumulation by improving photosynthetic efficiency.^25^, ^26^, ^27^

The AP673L spectrum's effectiveness can be attributed to its high red‐to‐blue (R:B) ratio and the inclusion of FR radiation, which may enhance light absorption by chlorophylls, improve balance excitation between PSI and PSII, and reduce photoinhibition under high irradiance conditions.^28^, ^29^, ^30^ This mechanism not only favors CO_2_ assimilation but also supports greater carbohydrate accumulation, which is directly related to DMP formation.^31^ Both radish cultivars showed stimulation of dry matter under the highest R:B ratio (5.5; AP673L lamp), which is consistent with other studies that demonstrate that biomass accumulation in *Brassica* microgreens is favored by red‐enriched and FR‐supplemented spectra.^26^, ^27^, ^32^ However, in some genotypes, like kohlrabi microgreens, FR addition decreased fresh and dry biomass,^26^, ^33^ indicating a strong species‐ and cultivar‐dependent response to FR supplementation.

Light intensity also played a crucial role in modulating agronomic outcomes. Overall, in our study, the highest intensity (300 μmol m^−2^ s^−1^) favored yield and dry matter accumulation in radish microgreens. This behavior was consistent with previous reports where higher intensity improved growth and biomass in *Brassica* microgreens.^14^, ^25^ However, it has been noted that the response is not always linear, as genotype and intensity thresholds influence yield, with reductions being observed under very low or very high irradiance conditions.^15^, ^34^, ^35^ In fact, in species such as cabbage, kale, beet, and broccoli, intermediate intensity levels have been found to be more favorable than extremes.^16^, ^21^ This highlights the need to consider species‐specific optimal when extrapolating results. In our case, the positive response to the highest intensity can be explained by its direct effect on photosynthesis, which increases proportionally with intensity until reaching saturation.^36^, ^37^ In turn, the photosynthetic process leads to biomass accumulation.^25^ Thus, the highest intensity would lead to improved biomass in radish microgreens of both cultivars by favoring this process.

LA, a key morphological trait defining market acceptance in microgreens,^38^ was also strongly influenced by the interaction of spectrum and intensity. The highest R:B ratio (5.5; AP673L lamp) promoted greater LA, especially at 200 μmol m^−2^ s^−1^ in green radish and at 300 μmol m^−2^ s^−1^ in purple radish, which likely contributed to improved light interception and photosynthetic capacity, reinforcing the biomass gains under this spectral condition. Besides, this was consistent with earlier studies showing that red‐ and/or FR‐enriched spectra promote leaf expansion.^26^, ^39^ In particular, the FR component has been shown to causes a shade‐like response, which could increase leaf expansion and reduce compensation points, ultimately enhancing net photosynthesis.^29^, ^30^ However, similar to yield responses, LA exhibited genotype‐specific patterns under varying intensities, with some Brassicaceae species showing reduced or no effect at higher intensities.^16^, ^40^ In our case, the consistent effect of the highest R:B ratio (5.5; AP673L lamp) suggests that spectral composition may modulate the intensity response, possibly dampening the reduction in LA often observed under bright light.^41^

### Influence of light spectrum and intensity on photosynthetic pigments

Chla and Chlb concentrations responded selectively to light treatments, with the highest R:B ratio (5.5; AP673L lamp) enhancing pigment content in purple radish microgreens. The intensity of 300 μmol m^−2^ s^−1^ also favored chlorophyll accumulation, particularly in green radish. These results support earlier observations that both spectrum and light intensity regulate chlorophyll biosynthesis via activation of key enzymes and gene expression pathways.^42^, ^43^, ^44^, ^45^, ^46^ Specifically, the results by Xie *et al*.^45^ showed that purple LED light exhibited the best effect on retaining chlorophyll via downregulating expression of genes related to chlorophyll degradation, including SGR, PAO, NYC1and RCCR. Meanwhile, Zhang *et al*.^47^ showed that more than half of the genes involved in the chlorophyll biosynthesis pathway were upregulated under the red–blue light in non‐heading Chinese cabbage, which may have contributed to the higher chlorophyll concentration in this species under red–blue light observed by Fan *et al*.^48^ Nonetheless, other wavelengths can also regulate other genes. For instance, Okamoto *et al*.^44^ indicated that key genes in chlorophyll biosynthesis (HEMA1, encoding the rate‐limiting enzyme glutamyl‐tRNA reductase, GSA, CHLH, and GUN4) could also be induced under white, blue, and red light. Conversely, Fan *et al*.^48^ observed that Chla and Chlb concentrations were lowest in non‐heading Chinese cabbage seedlings under red light, which may be due to lower biosynthesis of chlorophyll precursors, including ALA, Proto IX, Mg‐proto IX, and Pchlide. Thus, differences in the relative proportion of red, blue, and other wavelengths such as green or far‐red can modulate enzymatic activity and gene expression, leading to selective effects on Chla *versus* Chlb. Moreover, the response was cultivar‐specific, indicating that genetic background modulates the pigment response to environmental cues. For example, purple radish accumulated more Chlb under the highest R:B ratio (5.5; AP673L lamp), possibly due to enhanced activation of genes involved in Chlb biosynthesis, whereas green radish showed stronger responses of Chla to intensity changes. This indicates that red and green cultivars may differentially regulate gene expression and enzyme activity in response to identical light environments.

The dual role of intensity is also evident. While moderate‐to‐high intensities increased chlorophyll content in this study, intensities beyond a certain threshold may induce photo‐oxidative stress and pigment degradation, as previously noted in other studies.^43^, ^46^ Besides, some authors have observed that chlorophyll accumulation increases with intensity up to a certain limit, after which further increases reduce chlorophyll through degradation pathways.^49^, ^50^ In our study, the highest intensity (300 μmol m^−2^ s^−1^) favored chlorophyll concentrations, which may also explain the higher dry matter accumulation observed under these conditions. It should be noted that the biosynthesis of chlorophyll pigments by the intensity factor can be mediated by enzymatic regulations or metabolic signals. For instance, the results of Jones‐Baumgardt *et al*.^43^ indicated that the loss of Chla and Chlb in microgreens at high PPFDs is likely due to enzyme‐mediated degradation. On the other hand, Zhang *et al*.^46^ suggested that light intensity affects chlorophyll synthesis during the greening process via a metabolic signal, the AOX‐derived plastidial NADPH/NADP^+^ ratio change.

Carotenoid accumulation was significantly influenced by the interaction between spectrum and intensity. Spectra with a higher proportion of FR such as those with the highest R:B ratio (5.5; AP673L lamp), and lowest R:B ratio (1.9; NS12 lamp) promoted carotenoid synthesis, likely through enhanced expression of phytoene synthase (PSY), a key enzyme in the biosynthetic pathway.^51^, ^52^ In particular, the observed effect is supported by the knowledge that red and FR light stimulate PSY activity through phytochrome A signaling.^53^ In our study, the NS12 (low R:B ratio, 1.9) and AP673L (high R:B ratio, 5.5) lamps both had a comparatively low R:FR ratio, which is probably conducive to PSY expression and, as a result, carotenoid accumulation in both microgreens cultivars. Furthermore, these findings are consistent with studies showing that red, FR, and UV‐A wavelengths can stimulate carotenoid biosynthesis in various microgreen species.^10^, ^36^, ^38^

Interestingly, carotenoids also showed cultivar‐specific responses. The way that light signals affect transcription factors and carotenoid pathway enzymes is probably determined by genetic differences between green and purple radish. This may help to explain why the two cultivars' carotenoid accumulation levels varied even under the same spectral and intensity conditions.

### Influence of light spectrum and intensity on primary metabolites and GLS

Total soluble sugar concentration increased under the lowest R:B ratio (1.9; NS12 lamp) at 300 μmol m^−2^ s^−1^, a condition characterized by UV‐A and FR components. The promotion of sugar biosynthesis under this treatment is consistent with the enhancement of photosynthetic activity, leading to higher carbohydrate accumulation.^37^, ^54^ The correlation between increased pigment content, dry matter, and sugar levels under high intensity suggests that enhanced light capture and carbon assimilation drive the observed metabolic shifts.

Organic acid accumulation was also modulated by light conditions. In green radish microgreens, the lowest R:B ratio (1.9; NS12 lamp) combined with high intensity significantly increased total organic acids (TOAC), whereas in purple radish microgreens, the highest R:B ratio (5.5; AP673L lamp) was more effective. The presence of UV‐A and FR in NS12 lamp likely enhanced metabolic fluxes related to organic acid biosynthesis, as supported by previous findings.^55^, ^56^

TGC showed a marked cultivar‐dependent response. In green radish, the lowest R:B ratio (1.9; NS12 lamp) led to a higher TGC. On the other hand, in purple radish, the R:B ratio of 2.1 (Ph2.1 lamp) was most effective. The NS12 lamp was characterized by presenting UV‐A light in the spectrum and, according to He *et al*.,^57^ UV‐A appears to be a key factor in the activation of GLS biosynthesis genes, while the white‐light characteristics of Ph2.1 lamp may also promote TGC through broader photoreceptor activation.^19^ These patterns confirm that both spectral composition (presence of UV‐A, R:B balance, white component) and intensity modulate GLS biosynthesis and that the response is highly cultivar‐dependent, which is consistent with what has been observed in other Brassicaceae.^58^, ^59^ Previous studies have shown that UV‐A light can induce key genes of the GLS biosynthetic pathway such as *BCAT4*, *GGP1*, *SUR1*, *AOP2*, and *AOP3*,^57^ while white light would promote the expression of transcription factors such as MYB28‐2, MYB28‐3, and MYB29 in species of the same radish family as kale, stimulating the formation of GLS.^19^ Other wavelengths can also regulate GLS biosynthesis. For example, red–blue light (75%R + 25%B) upregulated key genes such as *MAM1*, *CYP83A1*, *UGT74C1*, *SOT17*, and *SOT18*, while red light alone mainly upregulated *CYP83A1*, *SOT17*, and *SOT18*. On the other hand, both spectra promoted the expression of *CYP79B2*, *CYP79B3*, and *CYP83B1*, which coincided with the increase in GLS in broccoli seedlings.^60^ In contrast, blue light can have a repressive effect by reducing the availability of precursors such as methionine or tryptophan and by inducing HY5, which inhibits *SOT18*, the GLS‐promoting gene.^60^ This could explain why different R:B ratios generate divergent GLS patterns in the two cultivars evaluated.

On the other hand, light intensity strongly influences GLS biosynthesis by regulating the expression of certain genes. Our results showed that intensities ≥ 200 μmol m^−2^ s^−1^ promoted GLS accumulation in green radish, whereas, in general, low to moderate intensities (100–200 μmol m^−2^ s^−1^) were more effective in purple radish under the same spectral conditions, highlighting a cultivar‐specific response. Similarly, Zhou *et al*.^58^ found that in two distinct cultivars of Chinese cabbage seedlings a light intensity of 200 μmol m^−2^ s^−1^ positively and differentially overexpressed key genes such as *BCAT4*, *MAM1*, *CYP79F1*, *CYP79B3*, *SUR1*, *UGT74B1*, and *UGT74C1*. Meanwhile, higher intensity (600 μmol m^−2^ s^−1^) activated a broader set of genes, including the transcription factor MYB28, linked to aliphatic and indole GLS biosynthesis, and that varied according to cultivar. These results collectively imply that genotype influences both the threshold and the level of gene activation, which could account for the different accumulation of GLS among cultivars in similar light conditions.

### Influence of light spectrum and intensity on EUE and EEE

EUE and EEE are relevant variables within a vertical farm system. The results obtained in green and purple radish microgreens show that EUE and EEE were influenced by spectral quality and, to a lesser extent, by the light intensity applied. The significant differences observed between the spectra indicate that light composition plays a decisive role in the conversion of electrical energy into biomass and in economic performance.

The lowest (1.9; NS12 lamp) and the highest R:B ratio (5.5) under the AP673L lamp presented significantly lower EUE and EEE values compared to Ph2.1, denoting superior energetic and economic performance, specially under AP673L lamp. The UV‐A fraction, observed in the NS12 lamp, can induce positive effects on the biomass of radish microgreens, especially when combined with far red.^26^ On the other hand, the AP673L lamp was characterized by a higher proportion of red and far red in the spectrum. Both wavelengths can promote hypocotyl length and cotyledon area in microgreens of some *Brassica* species^13^, ^30^, ^61^ – characteristics that positively influence higher yields. This is consistent with the higher yield and dry matter accumulation under the AP673L lamp. According to Tan *et al*.,^30^ far red can induce greater photosynthetic capacity by activating the expression of some genes such as *LHcb1* and *LHcb2* or by exciting photosystem I.

Light intensity also significantly affected both indicators. A clear downward trend in EUE and EEE was observed as light intensity increased from 100 to 300 μmol m^−2^ s^−1^. The lowest values for both variables were recorded at 300 μmol m^−2^ s^−1^, indicating that higher photon fluxes, although requiring greater total energy input, produce proportionally greater biomass, thus improving the energy‐to‐product ratio. Higher intensity correlated with greater accumulation of biomass, leaf area, and cotyledon weight in Brassicaceae microgreens^15^, ^39^ – variables that, when increased, improve EUE and EEE.

These results suggest that these spectra and intensities provide a more favorable balance between light absorption and energy consumption, possibly due to more efficient utilization of photons or reduced metabolic energy losses. However, purple radish microgreens consistently showed higher EUE and EEE values compared to green radish microgreens in all treatments, suggesting slightly lower energy efficiency. This could be related to the higher energy requirements associated with the synthesis of secondary pigments such as anthocyanins, which increase metabolic demand and may reduce overall energy conversion efficiency.

### CONCLUSIONS

This study demonstrates that both light spectrum and intensity play a crucial role in modulating the agronomic performance and metabolic composition of radish microgreens cultivated in a vertical farming system. Lighting optimization depended on the physiological or productive goal to be achieved. The AP673L lamp, which had the highest R:B ratio (5.5), rich in red and far‐red wavelengths, was particularly effective at 300 μmol m^−2^ s^−1^ to maximize yield and dry matter accumulation. In contrast, the most effective option for promoting the accumulation of primary metabolites, such as sugars and organic acids, was the NS12 lamp, specifically at 300 μmol m^−2^ s^−1^, which was distinguished by having a low R:B ratio (1.9), including UV‐A and an intermediate amount of far red. In addition, this lamp, together with the Ph2.1 lamp (R:B = 2.1), favored the accumulation of GLS. However, the choice of one lamp or another and its intensity depended on the radish cultivar to be grown. From a production standpoint, combining the AP673L spectrum with high light intensity (300 μmol m^−2^ s^−1^) resulted in the lowest EUE and EEE values, indicating the most favorable configuration in terms of energy and economic efficiency. Therefore, although spectral quality remains a determining factor in yield, optimizing light intensity is also essential for minimizing production costs and energy consumption. These findings underscore the potential of spectrum–intensity manipulation as a strategic tool to optimize both productivity and nutritional quality in microgreen production, offering practical applications for vertical farm systems.

## AUTHOR CONTRIBUTIONS

Conceptualization, methodology, and investigation: Martínez‐Moreno Flores, and Frutos. Data curation and formal analysis: Frutos, Navarro, and Martínez. Writing – original draft preparation: Martínez‐Moreno and Hernández‐Adasme. Writing – review and editing: Martínez‐Moreno, Hernández‐Adasme and Mestre. funding acquisition and project administration: Martínez.

## FUNDING INFORMATION

Funding was provided by Agencia Nacional de Investigación y Desarrollo (ANID) for the project Redes 190057 and Public–Private Partnership Projects program (CPP2021‐009146) by the Spanish Ministry of Science and Innovation (MCIN) and the State Research Agency (AEI) [10.13039/501100011033], with co‐funding from the European Union through NextGenerationEU/PRTR. Development of an automated unit for the sustainable production of high‐quality vegetables, EDEN (CPP2021‐009146), funded through the 2021 programme.

Different letters in the columns within each factor or interaction indicate significant differences (Fisher's LSD test, *P < 0.05).

## Data Availability

The data that support the findings of this study are available from the corresponding author upon reasonable request.
